# Double trouble: psoriasis and cardiometabolic disorders

**DOI:** 10.5830/CVJA-2017-055

**Published:** 2018

**Authors:** Goolam Mahyoodeen Nasrin, Tikly Mohammed, J Crowther Nigel

**Affiliations:** Department of Internal Medicine, Chris Hani Baragwanath Academic Hospital, Faculty of Health Sciences, University of the Witwatersrand, Johannesburg, South Africa; Department of Internal Medicine, Chris Hani Baragwanath Academic Hospital, Faculty of Health Sciences, University of the Witwatersrand, Johannesburg, South Africa; Department of Chemical Pathology, National Health Laboratory Services and University of the Witwatersrand, Johannesburg, South Africa

**Keywords:** psoriasis, cardiovascular disease, cardiometabolic disease, co-morbidities, metabolic syndrome, obesity

## Abstract

Psoriasis (PsO) is a chronic immune-mediated inflammatory skin disorder associated with numerous co-morbidities. This descriptive review focuses on the cardiometabolic co-morbidities of PsO with reference to the epidemiology and pathogenetic mechanisms linking PsO and cardiometabolic disease (CMD). Registry-based studies have shown PsO to be associated with an increased risk of cardiovascular morbidity and mortality. Factors linking PsO and CMD include: chronic inflammation, obesity, classic cardiovascular risk factors, and the effects of systemic therapy used to treat PsO. Chronic inflammation is associated with PsO itself, and with obesity. Adipose tissue is responsible for the secretion of various adipokines, which together with pro-inflammatory cytokines arising from the psoriatic plaque, contribute to the proinflammatory and pro-atherogenic environment. Systemic therapy aimed at decreasing inflammation has been shown to improve CMD in PsO. Screening for and treating CMD and initiating lifestyle modifications will remain the most important interventions until further data emerge regarding the effect of systemic therapy on CMD progression.

Psoriasis (PsO) is a complex, chronic, immune-mediated inflammatory skin disorder, which has a global prevalence ranging between 0.91 and 8.5%.^1^ It is recognised by the World Health Organisation as a major global health challenge,^2^ and is associated with impaired psychological quality of life, which exceeds that observed in several chronic conditions including malignancy and heart failure.^3^ A wide range of co-morbidities are associated with PsO, ranging from chronic inflammatory disorders such as inflammatory arthritis, [often referred to as psoriatic arthritis (PsA)],^4^ Crohn’s disease,^4^ neuropsychiatric disorders such as Parkinsonism,^5^ psychiatric disease,^6^,^7^ malignancies,^4^,^8^ as well as cardiometabolic diseases^9^-^13^ (Table 1).

**Table 1:** Co-morbidities associated with psoriasis

Psoriatic arthritis^4^ Crohn’s disease^4^ Parkinson’s disease^5^ Psychiatric disease - Major depression^6^ - Alcohol abuse^7^ Malignancy^4^,^8^ Chronic kidney disease^14^ Cardiometabolic diseases - Obesity15 - Metabolic syndrome^13^,^16^ » Type 2 diabetes^17^ » Hypertension^17^ » Dyslipidaemia^18^ - Myocardial Infarction^10^ - Stroke^19^,^20^ - Abdominal aortic aneurysms 21 - Non-alcoholic fatty liver disease^22^ Hyperuricaemia and gout 23

In this descriptive review, we examine the epidemiological and pathological evidence linking PsO and cardiometabolic disorders, with a particular focus on cardiovascular disease (CVD). We conducted a PubMed search using the term ‘psoriasis’ in combination with the terms ‘cardiovascular disease’, ‘co-morbidities’, ‘diabetes’, ‘metabolic syndrome’, ‘obesity’, ‘hypertension’, ‘dyslipidaemia’, ‘non-alcoholic fatty liver disease’ and ‘inflammation’. Our search was limited to articles published in English.

## Immunopathogenesis of psoriasis

The interplay between genetic factors and environmental triggers results in the classic psoriatic plaque, characterised histologically by epidermal hyperplasia, vascular hyperproliferation and chronic inflammation.^4^ Common triggers for the disease are local skin trauma (Koebner phenomenon), stress, Streptococcus pyogenes, infection and smoking.^4^ About a third of patients have a family history of PsO and genome-wide analysis studies have shown the PSORS1 gene, located on chromosome 6p, accounts for between 35 and 50% of the heritability of PsO.^24^

A complex cross-talk between keratinocytes and dendritic cells (DCs) in the skin, and T lymphocytes, mediated by a variety of cytokines, results in keratinocyte hyperplasia, the characteristic histological feature of PsO.^25^ Initially, keratinocytes recruit DCs to produce IL-23 and IL-12, which in turn activate mostly T helper (Th) 1 and Th17 cells, resulting in further up-regulation of cytokine secretion, especially IL-17, interferon-γ (IFNγ), tumour necrosis factor (TNF) and IL-22.^12^ These cytokines amplify psoriatic inflammation and keratinocyte hyperplasia.^4^ Moreover, pro-angiogenic factors produced by keratinocytes such as vascular endothelial growth factor drive abnormal vascular proliferation within the psoriatic plaque.^4^,^25^

## Psoriasis and cardiometabolic disorders

Recent interest has focused on the wide spectrum of cardiometabolic co-morbidities observed in subjects with PsO. These include obesity,^15^ the metabolic syndrome (MetS) and its components,^13^,^16^ non-alcoholic fatty liver disease (NAFLD)^22^ and CVD^10^,^19^,^20^,^26^-^28^ (Table 1). The dual burden of PsO and associated co-morbidities impacts negatively on health-related quality of life and is associated with an increased risk of premature death.^8^,^29^

There is now a large body of evidence linking chronic inflammation to accelerated atherosclerosis.^30^,^31^ Rheumatoid arthritis (RA) was the first chronic inflammatory condition to be associated with an increased risk of premature cardiovascular death, by as much as 50%.^32^ The traditional Framingham risk factors such as type 2 diabetes mellitus (T2DM), hypertension and smoking have been shown to only partly account for the increased cardiometabolic risk in RA.^33^

Mechanistically, the pro-inflammatory state of RA leads to cytokine-mediated accelerated atherosclerosis.^34^,^35^ The epidemiological evidence supporting the notion that PsO is associated with a similar increased CMD risk is less wellestablished and the precise pathobiology driving atherosclerosis in PsO is complex and not fully elucidated. One hypothesis is that the chronic inflammatory state of PsO triggers a ‘psoriatic march’ where the pro-inflammatory milieu leads to insulin resistance and endothelial cell dysfunction, predisposing to accelerated atherosclerosis, finally manifesting as clinical CMD.^36^

## Epidemiology of cardiometabolic disease in psoriasis 

The association of PsO with cardiovascular disease (CVD) was first proposed by McDonald and Calabresi in 1973 in a letter published in the New England Journal of Medicine.^37^ In a subsequent retrospective record review comparing 323 PsO and 325 non-psoriatic dermatology patients, they reported an increased prevalence of ‘occlusive vascular disease’, which included myocardial infarction (MI), stroke, thrombophlebitis and pulmonary embolism in PsO patients.^38^

Several recent registry-based studies, mainly from the United Kingdom (UK) and Denmark have supported these initial observations (Tables 2, 3). In a prospective study of 130 000 PsO patients and more than 500 000 control subjects in the UK General Practice Research Database (UKGPRD), PsO was found to be a risk factor for MI, with hazard ratios (HR) of 1.54 (95% CI: 1.24–1.91) and 7.08 (95% CI: 3.06–16.36) in mild and severe cutaneous PsO, respectively.^10^ The increased risk remained even after controlling for major cardiovascular risk factors such as age, gender, smoking, T2DM, hypertension, prior MI, dyslipidaemia and body mass index (BMI).

**Table 2:** Longitudinal studies arising from the UK General Practice Research database

*Reference*	*Sample Size*	*Control group size*	*Variables in multivariate models*	*Main outcomes*	*HR mild PsO*	*HR severe PsO*
Gelfand et al.^10^	Mild: 127 139 Severe: 3 837	556 995	Age. gender, HT, DM, BMI, previous MI, cholesterol, smoking	PsO may confer an independent risk of MI	1.54 (1.24–1.91)	7.08 (3.06–16.36)
Gelfand et al.^20^	Mild: 129 143 Severe: 3 603	Mild: 496 666 Severe: 14 330	Age, gender, HT, DM, cholesterol, smoking, cerebrovascular disease	Both mild and severe PsO were independent risk factors for stroke	1.06 (1.0–1.1)	1.43 (1.1–1.9)
Mehta et al.^51^	Severe: 3 603	14 330	Age, gender, smoking, DM, HT, Hyperlipidaemia	Severe PsO was an independent risk factor for cardiovascular mortality	–	1.57 (1.26–1.96)
Abubara et al.^52^	Severe: 3 603	14 330	Age, gender	Patients with severe PsO were at increased risk for death from CVD	–	1.57 (1.26–1.96)

HT, hypertension; DM, diabetes mellitus; BMI, body mass index; MI, myocardial infarction.

**Table 3:** Longitudinal studies arising from the Danish Nationwide cohort

*Reference*	*Sample Size*	*Control group size*	*Variables in multivariate models*	*Main outcomes*	*RR mild PsO*	*RR severe PsO*
Ahlehoff et al.^26^	Mild: 34 371 Severe: 2 621	4 003 625	Age, gender, co-morbidities, medication	Cardiovascular mortality was increased in patients with PsO	1.14 (1.06–1.22)	1.57 (1.27–1.94)
Ahlehoff et al.^26^	Mild: 34 371 Severe: 2 621	4 003 625	Age, gender, co-morbidities, medication	MI was increased in patients with PsO	1.22 (1.12–1.33)	1.45 (1.10–1.90)
Ahlehoff et al.^19^	Mild: 36 765 Severe: 2 793	4 478 926	Age, gender, co-morbidities, medication	PsO was associated with an increased risk of ischaemic stroke	1.25 (1.17–-1.64)	1.65 (1.33–2.05)

MI, myocardial infarction.

In the Danish studies of more than 36 000 PsO patients, PsO was associated with an increased risk of adverse CV events and all-cause mortality.^26^ Younger patients and those with severe cutaneous disease appeared to have the greatest risk, a finding that was similar to that for the UKGPRD cohort.^24^ Two meta-analyses, comprising 14 cohorts of patients from Europe and the United States have shown that the risk for CVD is greatest in patients with severe PsO.^27^,^28^ A major limitation of many studies included in the meta-analyses was the lack of adjustment for traditional cardiovascular (CV) risk factors.

Some smaller studies have failed to show the association between PsO and CVD. A prospective study of 1 380 PsO patients, followed up for 10 years, showed no increase in cardiovascular mortality (standard mortality ratio: 0.83, 90% CI: 0.7–1.0).^39^ In another study comparing over 15 000 patients and over 27 000 controls, PsO was found to be an equivocal risk factor for admission for ischaemic heart disease (IHD), after controlling for age and gender (HR = 1.10, 95% CI: 0.99–1.23).^40^ Moreover, age- and gender-matched survival analysis revealed no difference in the risk of acute MI.

A recent Dutch study of 262 ambulatory PsO patients over 55 years of age showed no increase in CV morbidity and mortality.^41^ The strength of this study was that CV events were identified using clinical data and specialised investigations, including CT scans, and electro- and echocardiography to minimise classification bias (all previous studies had used diagnostic codes). However, the limitations of this study were the small sample size, older age of the cohort (mean = 64 years), and that most patients had mild cutaneous PsO. Overall, the balance of evidence suggests that PsO, especially severe cutaneous disease, is associated with an increased risk of CVD.

Other metabolic disorders shown to occur more frequently in PsO are the MetS (and its components), hyperuricaemia, and non-alcoholic fatty liver disease (NAFLD). In a UK study, the MetS was more prevalent in PsO patients compared to the general population, more so in patients with severe than with mild cutaneous disease (OR for mild PsO = 1.22, 95% CI: 1.11–1.35; severe PsO = 1.98, 95% CI: 1.62–2.43).^13^ Furthermore, systematic reviews suggest that individual components of the MetS (dysglycaemia, obesity and hypertension) occur more frequently in PsO patients^15^,^42^,^43^ (Table 4).

**Table 4:** Systematic reviews showing association of type 2 diabetes, hypertension and obesity with psoriasis

		*Odds ratio*		
*Risk factor*	*No of Studies*	*Overall*	*Mild PsO*	*Severe PsO*
Type 2 diabetes^42^	27	1.59 (1.38–1.83)	1.53 (1.16–2.04)	1.97 (1.48–2.62)
Hypertension^43^	24	1.58 (1.42–1.76)	1.30 (1.15–1.47)	1.49 (1.20–1.86)
Obesity^15^	16	1.66 (1.49–1.89)	1.46 (1.17–1.82)	2.23 (1.63–2.05)

A meta-analysis of 16 observational studies found that PsO patients were more likely to be obese (pooled OR = 1.66, 95% CI: 1.17–1.82).^15^ A combined mean BMI of 30.6 kg/m^2^ has been observed in a review of clinical trials where biologics have been tested for the treatment of moderate to severe cutaneous PsO.^44^ Studies have also shown that PsO is associated with abdominal obesity, which is a proxy measure of visceral adipose tissue, and is a well-recognised risk factor for T2DM, hypertension, coronary artery disease and decreased life expectancy.^45^ The prospective Nurses’ Health Study II identified 809 incident cases of PsO and observed that it was more common in subjects with an increased waist circumference, a surrogate marker for abdominal visceral fat.^46^ In a cross-sectional case–control study, subjects with PsO were shown to have higher levels of visceral fat measured by computed tomography (CT) compared to controls.^47^ The role of visceral fat in chronic inflammation is discussed below.

Several studies have shown an increased prevalence of hypertriglyceridaemia in PsO patients.^13^,^16^,^18^ Comparisons of total cholesterol (TC) and low-density lipoprotein cholesterol (LDL-C) between patients and controls have yielded conflicting results. Some studies show an increase in TC and LDL-C levels in PsO patients compared to controls,^18^ whereas others show no significant differences or decreased levels.^48^ Asymptomatic hyperuricaemia is more common in PsO, even after correcting for confounders (age, gender and features of the MetS).^23^ Likewise, the risk of gout is also increased in PsO and PsA (HR = 1.71, 95% CI: 1.36–2.15).^49^

Several studies have demonstrated an increased prevalence of NAFLD in subjects with PsO.^22^ The diagnostic methods used in these studies included ultrasonography, biochemistry, transient elastography, liver biopsy, or combinations thereof. The prevalence of NAFLD was 65.6 versus 35% in matched controls (p < 0.001) when measured by ultrasonography.^50^

## Pathophysiological basis linking psoriasis with cardiometabolic disease 

**Inflammation: the common denominator**

There is now overwhelming evidence that chronic sub-clinical systemic inflammation accelerates atherosclerosis,^29^ including histological studies demonstrating the presence of inflammation in atherosclerotic lesions.^53^ Both innate and adaptive immunity are known to play a role in this process.^30^ This has been welldocumented in type 2 diabetes,^54^ and also in RA^34^ and systemic lupus erythematosus.^55^ Further evidence linking inflammation to atheroslcerosis is that C-reactive protein (CRP), a well-recognised biomarker of inflammation, is also associated with atherothrombotic disease, as well as the MetS and its components.^56^

As PsO is a systemic disease, the prevailing pro-inflammatory milieu is considered to contribute to the increased CMD risk.^57^ A meta-analysis of 78 studies found significantly elevated levels of pro-inflammatory cytokines, namely IL-6, TNF, CRP, E-selectin and ICAM 1, in PsO patients compared to controls.^58^

Obesity, which, as discussed previously, is prevalent in subjects with PsO, is a further source of inflammation. White adipose tissue (WAT), which is the primary component of visceral fat, is composed of both adipocytes and other immunologically active cells such as macrophages.^59^ Hence it is both metabolically and immunologically active. Adipocytes secrete adipokines, which are mainly pro-inflammatory, such as leptin, visfatin and resistin; as well adiponectin, which has anti-inflammatory properties.^59^ Other pro-inflammatory factors that are produced by WAT include TNF, IL-1, IL-6 and plasminogen activator inhibitor type 1 (PAI-1), all of which have been shown to directly or indirectly affect endothelial cell function and insulin sensitivity.^60^

## Possible genetic links

Epidemiological studies suggest a common genetic link between PsO, T2DM and obesity. In a 2016 cross-sectional, populationbased twin study in 33 588 Danish subjects, a significant association between PsO, T2DM and obesity was observed.^6^ Analysing data from twins discordant for PsO and including both monozygotic and dizygotic twin pairs, the study observed evidence for a shared genetic aetiology of obesity and PsO.

Moreover, there is evidence indicating that the strongest predictor of major adverse cardiovascular events (MACE) in patients with PsO is a family history of CVD.^62^ In a study of more than 25 000 mainly young Danes with mild PsO, and over 4 000 patients with severe disease, approximately two-thirds of patients in each group had a family history of CVD. The adjusted incidence rate ratios of MACE (with 95% CI) in patients with a family history of cardiovascular events were 1.28 (1.12–1.46) in those with mild PsO, and 1.62 (1.14–2.30) in subjects with severe PsO. There was no increased risk for MACE in PsO patients without a family history of CVD.

## Conventional cardiovascular risk factors

Patients with PsO have a higher burden of classic CV risk factors compared to the general population.^11^,^63^ In particular, and as discussed previously, there are higher prevalences of T2DM, dyslipidaemia, hypertension and obesity in subjects with PsO. Furthermore, it is known that patients with PsO have a higher prevalence of smoking than those who do not have PsO.^64^

## Drug therapy

Several drugs used to treat cutaneous PsO and PsA potentially increase the risk of cardiometabolic disorders. These include oral corticosteroids, cyclosporine and acitretrin, which may precipitate weight gain and unmask hypertension, T2DM and dyslipidaemia.^8^,^65^ Non-steroidal anti-inflammatory drugs, used in the treatment of PsA, increase the risk of hypertension and CVD.^66^

## Strategies to reduce cardiovascular risk in psoriasis

Psoriasis management guidelines highlight the importance of addressing cardiometabolic risk factors in PsO.^67^ There is a need for further research to address whether systemic therapy for PsO may potentially ameliorate CV risk.^67^,^68^

## General measures

Several expert groups recommend clinical and biochemical screening of PsO patients for early detection and management of co-morbidities such as hypertension, dysglycaemia and lipid abnormalities.^69^,^70^ The cessation of smoking, which is common in PsO,^71^,^72^ is critical in the management of this group of patients.^68^

Weight reduction not only reduces CV risk but has also been shown to attenuate the severity of PsO. A randomised, control study investigating the impact of hypocaloric dietary intervention and physical activity over 20 weeks in 303 overweight/obese patients with moderate to severe plaque PsO demonstrated a 48% reduction in PsO area and severity index score in the interventional arm, compared to only 25.5% in the informationonly arm (p = 0.02).73 The weight loss target of ≥ 5% was achieved in 29.8% of the interventional arm compared with 14.5% in the information-only arm (p = 0.001). There are also anecdotal case reports of improvement in PsO following bariatric surgery.^74^,^75^

## Drug therapy

**Anti-inflammatory agents used in the treatment of psoriasis**

Given the earlier discussion on chronic systemic inflammation as a risk factor for CMD, there is emerging evidence that controlling inflammation reduces the risk of CMD. There are several lines of clinical and biochemical evidence suggesting that methotrexate (MTX), a drug widely used for the treatment of moderate–severe cutaneous PsO and PsA, has anti-inflammatory and cardioprotective properties. MTX was found to provide a substantial survival benefit, mainly by reducing cardiovascular mortality rate in RA.^76^ The cardioprotective effect of MTX is mediated by increased adenosine levels that bind to adenosine A2 receptors on macrophages and block foam cell formation.^77^,^78^

In PsO, MTX and biologics such as the TNF inhibitors have been shown to lower the risk for CVD.^79^ Use of these agents in a Danish study have shown lower incidence rates of death, MI and stroke than patients on other therapies, with incidence (95% CI) rates of 6.0 (2.7–13.4) and 17.3 (12.3–24.3) for biologics and MTX, respectively, compared to 44.5 (34.6–57.0) for other therapies. Data from a small, prospective study and retrospective analyses suggest that systemic anti-inflammatory therapies (such as MTX or TNF blockers) in subjects with PsO or PsA may have the potential to ameliorate cardiovascular disease.^79^,^80^ While these have generated considerable interest, no conclusive data from randomised clinical trials are available to date.

***Impact of therapies for co-morbidities***

The anti-diabetic drugs pioglitazone^81^ and glucagon-like peptide 1 (GLP 1) agonists^82^ have been shown to improve PsO. Pioglitazone is a well-known insulin-sensitising agent, hence its efficacy may support the role of insulin resistance in the pathogenesis of PsO. The GLP 1 agonists are thought to be effective as a result of their extra-pancreatic effects, particularly their anti-inflammatory action.^83^ However, they may also exert indirect effects as their use is also associated with weight loss.

## Conclusions

A growing body of evidence supports the association between cardiometabolic diseases and PsO. The risk for CMD appears to be greatest with moderate–severe PsO and PsA. A complex interaction seems to exist between body adiposity, chronic inflammation and the psoriatic plaque, which leads to increased CVD risk.^57^ Screening and appropriate intervention, including lifestyle modification, to reduce the burden of cardiometabolic disease are important in the overall management and healthrelated quality of life of patients with PsO.
